# Structural and functional response of methane-consuming microbial communities to different flooding regimes in riparian soils

**DOI:** 10.1002/ece3.34

**Published:** 2012-01

**Authors:** Paul LE Bodelier, Marie-Jose Bär-Gilissen, Marion Meima-Franke, Kees Hordijk

**Affiliations:** Department of Microbial Ecology, Netherlands Institute of Ecology (NIOO-KNAW)Droevendaalsesteeg 10, 6708PB Wageningen, The Netherlands

**Keywords:** Flooding regime, floodplain, methane, methanotrophs, methane oxidation, PLFA, riparian, stable isotopes

## Abstract

Climate change will lead to more extreme precipitation and associated increase of flooding events of soils. This can turn these soils from a sink into a source of atmospheric methane. The latter will depend on the balance of microbial methane production and oxidation. In the present study, the structural and functional response of methane oxidizing microbial communities was investigated in a riparian flooding gradient. Four sites differing in flooding frequency were sampled and soil-physico-chemistry as well as methane oxidizing activities, numbers and community composition were assessed. Next to this, the active community members were determined by stable isotope probing of lipids. Methane consumption as well as population size distinctly increased with flooding frequency. All methane consumption parameters (activity, numbers, lipids) correlated with soil moisture, organic matter content, and conductivity. Methane oxidizing bacteria were present and activated quickly even in seldom flooded soils. However, the active species comprised only a few representatives belonging to the genera *Methylobacter*, *Methylosarcina,* and *Methylocystis,* the latter being active only in permanently or regularly flooded soils.

This study demonstrates that soils exposed to irregular flooding harbor a very responsive methane oxidizing community that has the potential to mitigate methane produced in these soils. The number of active species is limited and dominated by one methane oxidizing lineage. Knowledge on the characteristics of these microbes is necessary to assess the effects of flooding of soils and subsequent methane cycling therein.

## Introduction

Global change will result in increased frequency of flooding of riverine areas globally due to more intense precipitation events (Kleinen and Petschel–Held, 2007; Linde et al. 2011). Soils that traditionally have been dry terrestrial soils often used for agricultural purposes will be subjected to flooding events provoking large chemical, physical as well as biogeochemical changes in these systems (Hamilton 2010). One of the consequences of flooding may be that dry soils turn from a sink into a source of atmospheric methane which is, next to CO_2,_ the most important greenhouse gas adding about one-third to the radiative forcing exerted by CO_2_ (Denman et al. 2007). This will largely depend on the presence and functioning of methanogenic and methanotrophic microbial communities (Conrad 2007). It has been demonstrated that in concert with increasing methane production, the diversity of the methanogenic community increased substantially in line with the flooding frequency (Kemnitz et al. 2004). With respect to the methane-consuming community, however, there is hardly any information available regarding their response to different flooding regimes in irregular flooded soils.

Aerobic methane-oxidizing bacteria (MOB) play a vital role for global warming issues as they are the only biological sink for the greenhouse gas methane under aerobic conditions (Hanson & Hanson 1996; Conrad 2007). In dry upland soils (e.g., forest, grassland), they account for approximately 6% of the global sink strength of atmospheric methane and in wetlands they attenuate the source strength by 10–30% (Denman et al. 2007; Dunfield 2007). MOB utilize methane as carbon and energy source and belong to two phyla: the Proteobacteria and the Verrucomicrobia (Semrau et al. 2010). The latter have only recently been discovered (Dunfield et al. 2007; Pol et al. 2007; Islam et al. 2008) in extreme habitats. The proteobacterial MOB have been studied more extensively, and there are now 15 recognized genera within the alphaproteobacterial and gammaproteobacterial classes of this phylum (Bowman 2006; Conrad 2007; Rahalkar et al. 2007; Iguchi et al. 2010). MOB in these classes are often referred to as type I (Gammaproteobacteria) and type II (Alphaproteobacteria). This division is based not only on phylogeny (Gamma- vs. Alphaproteobacteria) but also on their carbon fixation pathway (ribulose monophosphate vs. the serine pathway), the arrangement of internal membrane stacks (perpendicular vs. parallel to the cell envelope), and phospholipid fatty acid profiles (PLFA) (Hanson & Hanson 1996; Bowman, 2006; Bodelier *et al*. 2009). A very distinct characteristic of these bacteria is the presence of specific PLFA that differentiates them from each other (type I: C16:1ω8c and C16:1ω5t vs. type II: C18:1ω8c) but also from all other organisms (Bodelier et al. 2009). Tracing the C of methane (which they use as only carbon and energy source) back into these PLFA by using stable isotope (^13^C) (Boschker et al. 1998; Nold et al. 1999) or radioisotope (^14^C) (Bodelier et al. 2000) labeling enables to link the biogeochemical function of these bacteria with their phylogeny and community composition. Activity and distribution of MOB is regulated mainly by the availability of oxygen, methane, and the presence of mineral nitrogen (Hanson & Hanson 1996; Bodelier and Laanbroek 2004). All these factors can change when a soil is flooded (Bodelier, 2003). The presence of plants can in concordance with flooding drastically influence the dynamics of oxygen, methane, and mineral nutrients (Bodelier, 2003; Bodelier et al. 2006; Laanbroek 2010).

The aim of the present study is to assess the function, diversity, and the link between these parameters in methane-consuming microbial communities in response to different flooding intensities. The MOB community is investigated using activity assays, Most Probable Number (MPN) counting, 16S rDNA-DGGE, PLFA, and stable isotope labeling of PLFA (SIP-PLFA) in four sites differing in flooding history within the catchments of the river Rhine.

## Materials and Methods

### Description of sampling site

**Figure A1:**
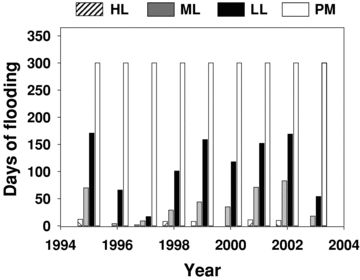
Number of days that the sites are flooded per year.

The sites where chosen on the basis of their flooding history and were all located along the river Waal (i.e., contributory of the river Rhine) and have been described earlier (Kemnitz et al. 2004; Steenbergh et al. 2010). Three sites were situated near the village of Ewijk (N 51° 52′ E 05° 53′), The Netherlands. The sites HL (high level), ML (mid-level), and LL (low level) differed in elevation with respect to the river and in the number of flooded days per year (see supporting information Fig. A1). The sites are hydrologically isolated, which means that it is only influenced by the river and not by groundwater seepage from surrounding areas. The site is subjected to extensive grazing all year round (1 head of cattle per 3–4 ha^–1^). Elevation of HL is 10.75 m above sea level. The soil type is clay-loam and the dominant vegetation type is *Lolium perenne/Ranunculus acris.* The ML site is located 8.90 m above sea level with silty-clay-coam soil and *Alopecurus pratensis/Ranunculus repens* as dominant vegetation. The elevation of the LL site is 7.26 m above sea level. The soil type differed with the depth profile: 0–5 cm = Sandy-loam; 5–10 cm = Loam; 10–20 cm = Silt-Loam. The site is vegetated by pioneer vegetation consisting of *Rumex palustris/Plantago major int.* The three irregular flooded sites were compared with an almost permanently flooded marsh located near Nijmegen, The Netherlands. The PM (i.e., permanent marshland) site at the “Oude Waal” (N 51° 51’ E 05° 53’) is an oxbow lake being a former river arm. The site is described in detail (Brock et al. 1987). The emergent macrophyte zone where the samples were taken is subjected to water level fluctuations. During winter and spring, the site is completely flooded due to water inlet from the river. The water inlet can be regulated by the local managers. In the course of the summer however, the water level is lowered due to seepage and evaporation. This can lead to periods during which the emergent macrophytes grow under waterlogged but not flooded conditions. Elevation of PM is <9 m above sea level (not determined) with a silt-Loam soil type dominated by the emergent macrophyte *Glyceria maxima*.

The physico-chemical characteristics of the four sites can be found in Tables A1 and A2.

### Sampling and sample processing

Soil samples were collected in winter (December 1999), spring (May 2000), and autumn (September 2000). Soil samples were taken at HL, ML, LL, and PM within a 1 m^2^ plot where four replicate soil cores were taken randomly (length 20 cm, diameter 3.8 cm). Immediately upon arrival in the laboratory, the cores were sectioned in layers (0–5 cm, 5–10 cm, 10–20 cm). Roots were removed from the soil and after homogenization and sieving (2 mm) samples were used for methane oxidation assays. Soil portions for MPN and chemical analyses were stored at 4°C, whereas soil for molecular analyses was stored at –20° C.

### Sediment physicochemistry

Sediments subsamples used for physico-chemical measurements were dried at 40 °C and analyzed to measure pH (extracted in water and in CaCl_2_),%CaCO_3_,% organic matter, total N, total and available P, Na^+^, K^+^, Mg^2+^, Fe^3+^, Cl^–^, SO_4_^2–^, NH_4_^+^, NO_3_^–^, and conductivity all according to (Troelstra et al. 1995). Separate subsamples were used to determine water content (weight loss of 5 g sediment samples dried at 105 °C for 24 h).

### Potential methane oxidation activity and ^13^C-PLFA labeling

The potential CH_4_ oxidation activity of the soil was determined as described by (Bodelier and Frenzel 1999). In short, 10 g of soil was transferred to 150-mL flasks and diluted 1:1 (w/v) with sterile demineralized water. The flasks were closed with rubber stoppers and 1.3 mL pure CH_4_ (10,000 ppmv) was added. Half of the samples were incubated with 20 µL 99.9%^13^C-CH_4_ (Campro Scientific, Wageningen, The Netherlands) elevating the δ^13^ CH_4_ signature to approximately +1400‰. The other half served as unlabeled control. Lipid analyses and GC-IRMS (Gaschromatography coupled to Isotope ratio masspectrometry) analyses of individual lipids were performed as described below. The flasks were incubated on a shaker (150 rpm) at 20 °C. The decrease of CH_4_ in the headspace was monitored by GC analysis of regularly taken subsamples. Since methane depletion curves typically display biphasic kinetics, an initial and an induced depletion rate can be distinguished (Steenbergh et al. 2010). The initial rate indicates active methanotrophic biomass at time of sampling, whereas the induced rate is a measure for total activatable methanotrophic abundance in the sample (i.e., including bacteria that were not active *in situ*) (Gilbert and Frenzel 1998). The potential methane oxidation rates (initial and induced) were calculated using the slope of the regression analyses on the linear parts of the methane depletion curves, where for the initial activity a timeframe was followed (15 h) which excluded possible growth of the methanotrophs (Steenbergh et al. 2010).

### Most probable numbers of methanotrophs

The number of methane oxidizers was determined by means of the MPN method according to (Bodelier and Frenzel 1999). In order to extract the cells from the sediment particles, 10 g of soil was diluted 1:5 with Phosphate buffered saline (PBS). The extracts were shaken for 4 h at 150 rpm and subsequently diluted 1:10 with PBS. A subsample of 100 µL of the suspension was serially diluted 1:1 in sterile microtiter plates (Nunc™ Brand Products, Denmark) containing growth medium for methanotrophs (NMS). The plates were incubated for 4 weeks at 25 °C in gastight jars containing 20% methane in air. Inoculated plates without methane served as controls. Wells that were turbid were considered positive. MPNs were obtained from statistical tables (Rowe et al. 1977).

## PLFA and ^13^C-PLFA Analyses

### Lipid analyses and Stable Isotope Probing (SIP-PLFA) of lipids

The *in situ* soil from May as well as the slurry samples at the end of the methane activity assay were subjected to PLFA analyses as described by (Mohanty et al. 2006) using 4 g of freeze-dried soil with a Bligh and Dyer extraction procedure as modified and described previously (Boschker et al. 1998) (Boschker et al. 2001).

### MOB cell numbers from PLFA

To calculate cellnumbers of type I and II MOB, PLFA were transformed to cell numbers as described previously (Sundh et al. 1995; Mohanty et al. 2006). For type I, it was assumed that 33% of the total PLFA content of the cells is C16:1ω8c; a cell contains 100 µmol PLFA · g^–1^ dry cells, using a conversion factor from dry weight to number of cells of 0.119×10^12^. For type II, it was assumed that 49% of the total PLFA content is C18:1ω8c, using a conversion factor from dry weight to cell numbers of 0.075× 10^12^.

## PCR-DGGE Analyses of MOB Communities

### DNA isolation

DNA from cultures and soil samples was extracted using a bead-beating protocol as described earlier. (Henckel et al. 1999). DNA from soil samples was repurified using Wizard DNA clean up columns (Promega, Madison, WI, USA).

### PCR amplification

PCR (polymerase chain reaction) of the 16S rRNA was performed as described earlier using a nested protocol (Bodelier et al. 2005). Strategy number 2 was used for amplification of type I MOB and strategy nr. 9 for type II. PCR amplification was performed in an MBS 0.5 S thermocycler (ThermoHybaid, Ashford, UK) in a 25 µL reaction mixture containing approximately 25 ng of DNA, 10 mM Tris/HCl pH 8.3, 50 mM KCl, 0.04% w/v Bovine Serum Albumin, 200 µM of each deoxynucleotide, 1.5 mM MgCl_2_, 25 U/mL of *Taq* DNA polymerase and 0.5 µM of each primer. In case of nested PCR designs, depending on the strength of the PCR product in the first round, either 5 µL of undiluted PCR product was used as a template for the second round or 5 µL of 10 or 100 times diluted first round PCR product.

### DGGE profiling

DGGE was performed indentical as described earlier (Bodelier et al. 2005).

### Sequencing and sequence analysis

To identify DGGE bands, a small piece of gel from the middle of the target band was excised from the DGGE gel using a sterile scalpel and incubated in 50 µL sterile milli-Q purified water for 24 h at 4°C. After this period, the DNA has diffused out of the agarose and the solution can be used as template in a re-amplification PCR. Re-amplification was performed using the original primers and PCR programs and run on DGGE to confirm its identity. Only pure bands were used for sequencing by amplifying with primers without a GC-clamp. PCR products for sequencing were purified and sequenced using Applied Biosystems 3730 and 3100 genetic analyzers by Baseclear Labservices (Baseclear, Leiden, The Netherlands).

Partial 16S rRNA sequences were compared with the sequences available in public databases, using the BLAST software from the National Center of Biotechnology Information (http://www.ncbi.nlm.nih.gov/BLAST/), to determine their phylogenetically closest relatives. Sequences have been submitted in the NCBI database under accession numbers JN254762-JN254781. Sequences were aligned to related sequences available in the public databases using the ARB software (Ludwig et al. 2004). Phylogenetic trees were calculated and drawn using the Neighbor-Joining algorithm using Jukes-Cantor correction as implemented in the TREECON software (Vandepeer and Dewachter 1994). For tree construction, only the aligned positions from the DGGE bands were compared with the sequences from the database.

## Statistical Analyses

### Effects of site, sediment depth, and season

Effects of sampling site (i.e., flooding regime), soil depth, and season on measured parameters were tested using factorial three-way analysis of variance (ANOVA) analyses. Correlations between measured variables were analyzed using the Spearman Rank correlation test. Previous to all ANOVA analyses these data were checked for normality (plots of SD vs. means) and were checked for homogeneity of variances (Levene's test). If necessary these data were transformed to meet the assumptions of the ANOVA analyses. All analyses were performed using the STATISTICA software package version 6.1 (Statsoft Inc., Tulsa, US).

### Multivariate analyses of SIP-profiles

Stable Isotope Probing ^13^C-PLFA profiles of slurry samples were compared to profiles of methanotrophic cultures to identify active MOB using multivariate analyses as described earlier (Mohanty et al. 2006; Deines et al. 2007).

## Results

### Root biomass

Root biomass increased significantly with increasing flooding intensity, decreased with depth in the soil, and did not differ between seasons (Table A3).

### Methane oxidation activity

**Figure 1:**
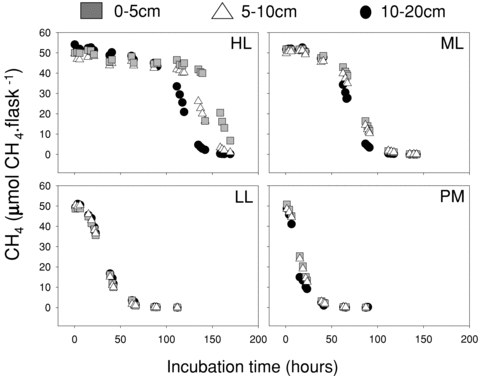
Examples of methane depletion curves in methane oxidation assays as recorded in December 1999. It is obvious that at the sites HL, ML, and LL there is a clear phase of initial oxidation followed by accelerated (“induced”) methane oxidation. In the PM samples, oxidation proceeds linear from the start to the end of the incubation.

**Figure 2:**
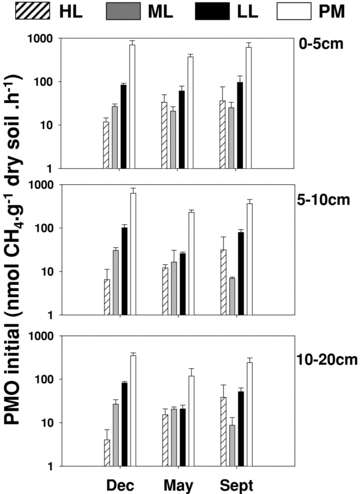
Initial methane oxidation rates as assessed in winter, spring, and autumn in four plots covering differences in flooding regime. Cores were subsectioned into three soil layers (0–5 cm, 5–10 cm, and 10–20 cm). Bars represent the mean ± standard deviation of four replicate samples per site.

The onset of methane initial consumption differed according to the flooding regime. In HL and ML sites it took about 24 h before any detectable methane consumption took place (Fig. 1). In LL and PM, there was immediate activity. The induced phase started in the HL site only after 120 h while this was much shorter in ML, LL, and even immediate in PM (Fig. 1). When expressing the initial methane consumption rates per gram of dry soil per hour, it is evident that methane oxidation rates increased with increasing number of days of flooding (Fig. 2). This is very clear in December. In May and September, the activity in the ML site was lower than HL and LL. Analyzing the initial as well as induced methane consumption rates statistically revealed that methane consumption increased significantly in more frequently flooded sites, it decreased with depth in the soil and it is higher in winter (Table A3). There were also significant interactions among the variables site, depth, and season (Table A3).

### Most probable numbers of methane oxidizers

**Figure 3:**
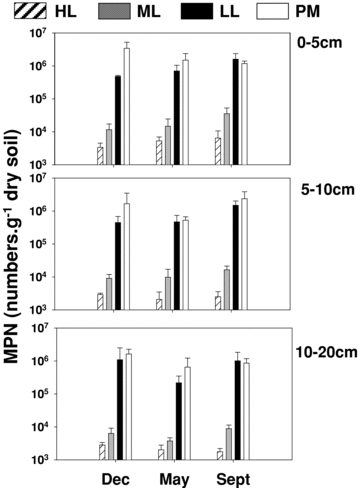
Most probable number counts of methanotrophic bacteria as assessed in winter, spring, and autumn in four plots covering differences in flooding regime. Cores were subsectioned into three soil layers (0–5 cm, 5–10 cm, and 10–20 cm). Bars represent the mean ± standard deviation of four replicate samples per site.

The numbers of methane oxidizers as determined using MPN counting displayed more or less the same trend as methane oxidation rates (Fig. 3). Numbers significantly increased with increased flooding frequency (Table A3) and were generally higher in winter (Fig. 3 and Table A3) but did differ between different soil layers.

### Group specific MOB abundance using PLFA analyses

**Figure 4:**
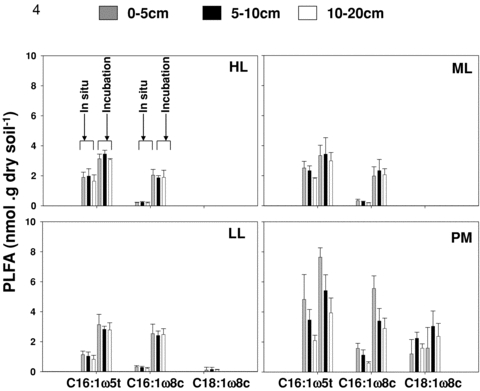
Abundance of PLFA (phospholipid-derived fatty acids) specific for methanotrophic subgroups (C16:1ω5t and C16:1ω8c for type I MOB, C18:1ω8c for type II MOB) as derived from soil cores sampled May 2000 in four plots covering differences in flooding regime. Cores were subsectioned into three soil layers (0–5 cm, 5–10 cm, and 10–20 cm). Bars represent the mean ± standard deviation of four replicate samples per site. Depicted are bars indicating PLFA abundance as directly extracted from field samples (*in situ*) and PLFA extracted from slurries at the end of methane oxidation assays (incubation).

**Figure 5:**
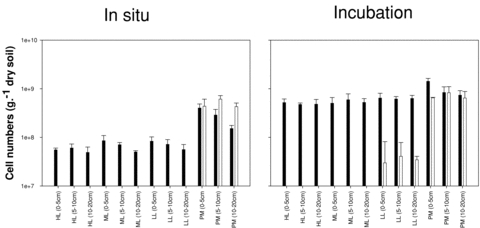
PLFA-based calculated **c**ell numbers (see materials and methods) of type I (black bars) and type II (white bars) MOB as derived from soil cores sampled May 2000 in four plots covering differences in flooding regime. Cores were subsectioned into three soil layers (0–5 cm, 5–10 cm, and 10–20 cm). Bars represent the mean ± standard deviation of four replicate samples per site. Depicted are bars indicating PLFA abundance as directly extracted from field samples (*in situ,* left panel) and PLFA extracted from slurries at the end of methane oxidation assays (incubation, right panel).

Type specific PLFA (i.e., C16:1ω5t, C16:1ω8c for type I and C18:1ω8c for type II) were analyzed in May immediately from the soil (i.e., *in situ*) or after incubation with 1% CH_4_ (i.e., incubation) (Fig. 4). It is evident from Figure 4 that type I PLFA predominated all sites, seldom flooded soils as well as permanently flooded sites. Type II PLFA was only detected in the permanently flooded freshwater marsh (Fig. 4) and also after incubation in the LL soil. Even after incubation with 1% CH_4,_ no type II PLFA was detected in HL and ML soil. Statistical analyses revealed that type I PLFA (i.e., *in situ* as well as after incubation) increased with flooding intensity (i.e., site) and decreased with depth (Table A3). Interestingly, type II PLFA did not decrease with depth in the PM site (Fig. 4). Based on the specific PLFAs, MOB cell numbers were calculated as depicted in Figure 5. In general, these numbers were higher than MPN counts of the same samples. Type I MOB increase in all sites during incubation, whereas type II increases only in LL. No growth of type II is observed in HL and ML. Type II only outnumbers type I in the PM site below 5 cm. After incubation type I and II reached equal numbers in the PM site.

### Stable Isotope Profiling of PLFA (^13^CH_4_–SIP PLFA)

**Figure 6:**
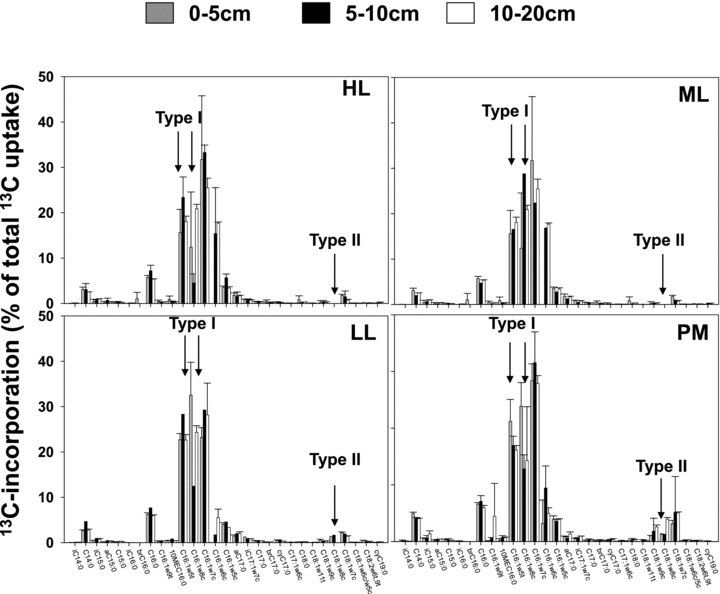
Incorporation of ^13^C-CH_4_ into PLFA in soil slurries derived from soil cores sampled May 2000 in four plots covering differences in flooding regime. Cores were subsectioned into three soil layers (0–5 cm, 5–10 cm, and 10–20 cm). Bars represent the mean ± standard deviation of four replicate samples per site. MOB type specific PLFA are indicated by an arrow.

**Figure A2:**
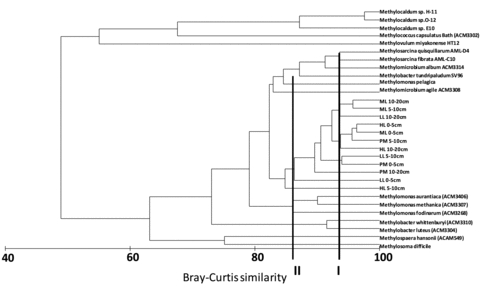
Type I MOB Cluster analyses (see Materials and methods for description) of ^13^C-CH_4_ labelling profiles of soil cores sampled in May 2000 in 4 plots covering differences in flooding regime. Cores were sub sectioned into 3 soil layers (0–5cm, 5–10cm and 10–20cm) before labelling incubations. The profiles depicted for every sample is the average profile of 4 replicate cores per sampling site. The bar labelled as I indicates the nearest cultured representative (*Methylosarcina*) of 3 clusters of soil samples (ML10–20cm+ML5–10cm+LL10–20cm; HL0–5cm+ML0–5cm+PM5–10cm+HL10–20cm; LL5–10cm+PM0–5cm). Bar II indicates that *Methylobacter tundripaludum* SV96 is the nearest cultured representative of the samples LL0–5cm+HL5–10cm.

**Figure A3:**
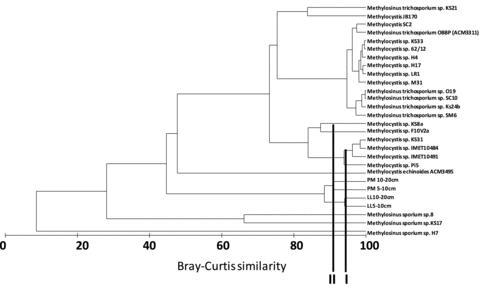
Type II MOB Cluster analyses (see Materials and methods for description) of ^13^C-CH_4_ labelling profiles of soil cores sampled in May 2000 in 4 plots covering differences in flooding regime. Cores were sub sectioned into 3 soil layers (0–5cm, 5–10cm and 10–20cm) before labelling incubations. The profiles depicted for every sample is the average profile of 4 replicate cores per sampling site. The bar labelled as I indicates the nearest cultured representative (*Methylocystis KS31* cluster) of the soil samples LL5–10cm+LL10–20cm. Bar II indicates that *Methylocystis KS8a* and F10V2a is the nearest cultured representative of the samples PM5–10cm+PM10–20cm.

Figure 6 displays the incorporation of ^13^C-CH_4_ into PLFA in potential methane consumption assays. Incorporation into individual PLFA's is expressed as percentage of the total ^13^C incorporation in all PLFA's analyzed. In HL and ML soils, the label is exclusively incorporated into PLFA that can be associated with type I MOB (i.e., C14:0, C16:0, C16:1ω7c, C16:1ω5t, C16:1ω6c, C16:1ω5c). Only in LL and PM soils a minor amount of label is detected in type II MOB specific PLFA (i.e., C18:1ω8c). The SIP-^13^C-CH_4_ profiles can be compared to PLFA profiles from cultured MOB (see Methods section) to find the most similar MOB species to the observed labeling pattern. Using multivariate statistics (Cluster analyses) similarities between profiles from soils and cultures can be established. This has been performed for all soil samples and is presented in the supporting information (Figure A2 for type I and Figure A3 for type II MOB, respectively). For type I, basically two groups of samples can be distinguished with highest similarity to cultured representatives. Group I (ML10-20 cm + ML5-10 cm + LL10-20 cm; HL0-5 cm + ML0-5 cm + PM5-10 cm + HL10-20 cm; LL5-10 cm + PM0-5 cm) have highest similarity with representatives of the genus *Methylosarcina*. Group 2 (LL0-5 cm + HL5-10 cm) are most similar to *Methylobacter tundripaludum SV96*. No clear clustering of the soil samples was observed due to site or depth. For type II MOB, (Fig. A3) label incorporation was detected only for the LL and PM soil that were most similar to *Methylocystis* species.

### 16S-rRNA DGGE profiling and phylogenetic assignment

**Figure 7:**
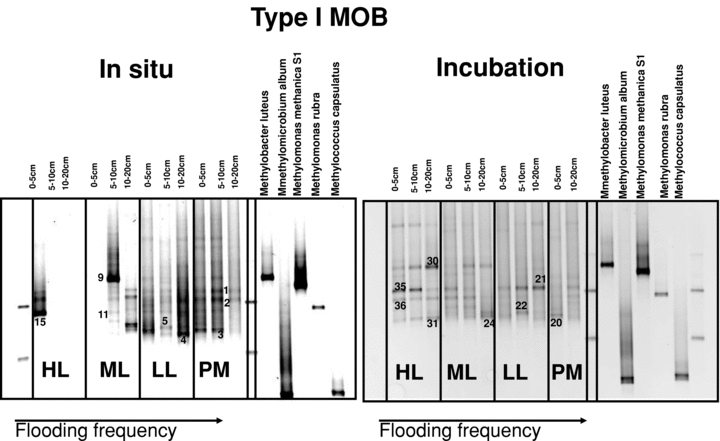
16S rDNA-based DGGE assay targeting type I MOB. DNA was extracted from soil cores sampled in May 2000 in four plots covering differences in flooding regime. Cores were subsectioned into three soil layers (0–5 cm, 5–10 cm, and 10–20 cm). Depicted are DGGE profiles as derived from soil directly coming from the field (*in situ,* left panel) and as obtained from DNA extracted from slurries at the end of methane oxidation assays (incubation, right panel). Bands are numbered and correspond to the sequences as presented in Table A5 as well as Figure 9.

**Figure 8:**
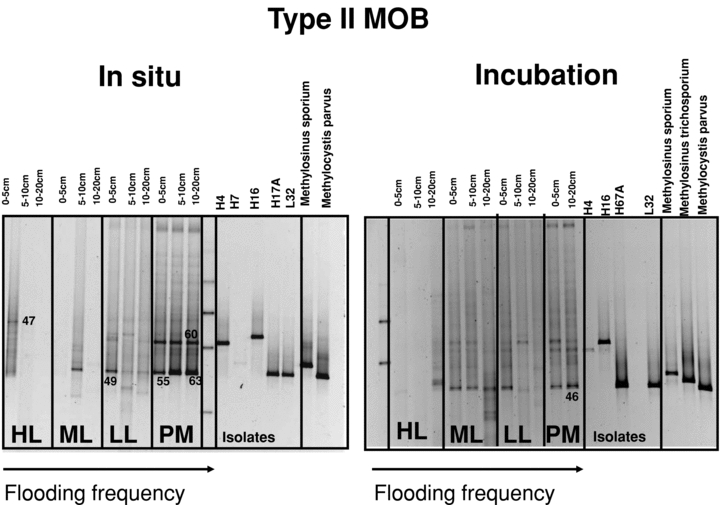
16S rDNA-based DGGE assay targeting type II MOB. DNA was extracted from soil cores sampled in May 2000 in four plots covering differences in flooding regime. Cores were subsectioned into three soil layers (0–5 cm, 5–10 cm, and 10–20 cm). Depicted are DGGE profiles as derived from soil directly coming from the field (*in situ,* left panel) and as obtained from DNA extracted from slurries at the end of methane oxidation assays (incubation, right panel). Bands are numbered and correspond to the sequences as presented in Table A5 as well as Figure 10.

**Figure 9:**
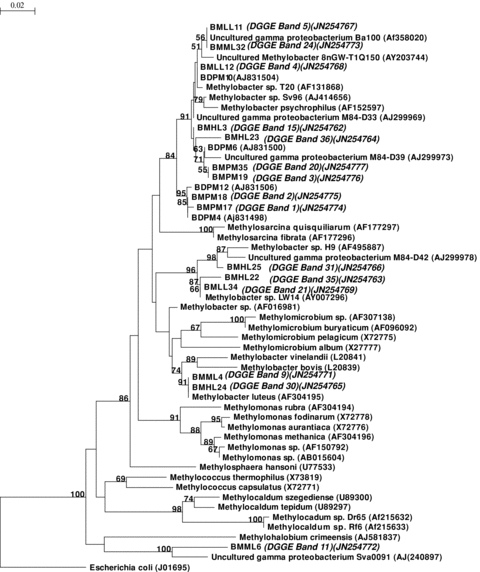
Phylogenetic tree based on 16S rRNA gene sequences (E. coli positions 555–926) showing the relationship of DGGE bands of type I MOB (Gammaproteobacteria) for the DGGE gel displayed in Figure 7 of the main manuscript with the most closely related members of the gammaproteobacteria. Bootstrap values (percentages) greater than 50% are represented at the nodes (1000 replicates).

**Figure 10:**
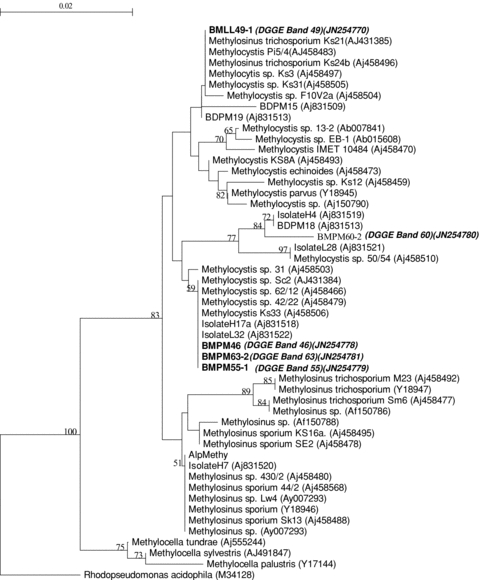
Phylogenetic tree based on 16S rRNA gene sequences (E. coli positions 555–926) showing the relationship of DGGE bands of type II MOB (Alphaproteobacteria) from Figure 8 of the main manuscript with the most closely related members of the alphaproteobacteria. Bootstrap values (percentages) greater than 50% are represented at the nodes (1000 replicates).

It was not possible to get PCR products for all samples, probably due to the low target number. Therefore, firm statements about the effect of the flooding regime on the *in situ* community profiles cannot be made. The type I MOB profiles of the *in situ* samples were rather similar in ML, LL, and PM and contained eight different bands (Fig. 7) that could be sequenced. After incubation, PCR products were readily obtained from the HL and ML sample that was not possible before incubation. It was obvious that the profiles from HL and ML after incubation were more diverse as compared to LL and PM. Next to this, LL and PM samples were less diverse after incubation. All retrieved sequences were most similar to MOB of the genera *Methylobacter*, *Methylosarcina,* and *Methylomicrobium* (Table A5 and Fig. 9). For type II MOB, good DGGE patterns for *in situ* soil samples were only obtained from LL and PM that were rather similar consisting of two main bands (Fig. 8). After incubation, PCR product was also obtained for ML, which displayed a similar pattern as in LL and PM, which contained and additional band as compared to the *in situ* samples (Fig. 8). All type II sequences were related to *Methylocystis* species (Table A5 and Fig. 10).

### Factors related to structure and response of methanotrophic communities

Since soil physico-chemistry was only analyzed in December, a correlation analyses was performed with the data from that month (Table A4). Methane oxidation activity (initial as well as induced) and MPN numbers were highly positively correlated with moisture%, conductivity, and organic matter%. The same factors were negatively correlated with pH and CaCO_3_%. Numbers and activities were also positively correlated with total N, Na, K, Mg, Mn, and Cl. There was a negative correlation with Cu. PLFA were only analyzed in May and were correlated with other variables measured in that month (Table A6). Methane oxidation and MPN numbers correlated positively with root biomass. Activity also correlated with MPN numbers. Methane oxidation activity correlated with all MOB PLFA data of type I specific lipids, whereas MPN only correlated with C16:1ω8c. There were a few parameters that were measured in all sampled months (Table A7). These analyses revealed that initial as well as induced oxidation correlated with root biomass and moisture%. However, moisture appeared to be the most strongly influencing factor.

## Discussion

Predicting effects of climate change on ecosystem functioning is one of the most pressing research needs in ecology in general. Especially assessing and understanding feedbacks is of utmost importance. Changes in temperature, precipitation and nitrogen deposition, and associated changes in vegetation can all influence microbes producing or consuming methane, thereby mediating positive or negative feedbacks to the climate system (Singh et al. 2010). The present study clearly demonstrates that increased flooding of soils will lead to a more responsive methane oxidizing community with higher abundances and activity potentials to mitigate the expected higher methane emission (Hamilton 2010) from these soils. However, the structure of the community will be a determinative factor, just as the environmental physico-chemistry. Although the state of the art methodology and the nature of the soil at the time this study was carried did not allow us to perform soil methane availability and methane flux measurements that could be directly coupled to activity and diversity of MOB, the results as described below are still of great value in anticipating effects of flooding on methane cycling in riparian soils.

Soil moisture, organic matter, and conductivity all had strong positive correlations with MOB activity and abundance. Moisture (i.e., water table) and organic matter content are well known regulating factors being directly connected to soil oxygen penetration and methanogenesis (Kettunen 2003; Bodelier et al. 2006; Conrad 2007). In a parallel study, for the same sites it was demonstrated that indeed, methane production potential increased in these sites and was high in LL and PM and not detectable in HL and ML (Kemnitz et al. 2004). The high moisture content in the PM site (on average 58%) would restrict oxygen coming into the soil, which is also most of the year flooded. However, the samples were taken in the littoral zone within a *Glyceria maxima* stand that introduces oxygen by its roots thereby facilitating methane oxidation, as has been demonstrated for other emergent aquatic macrophytes (Vandernat et al. 1997; Bodelier et al. 2000; Frenzel 2000; Siljanen et al. 2011).

The strong correlation with conductivity is less easy to explain. Stimulatory effects of mineral nitrogen (Bodelier et al. 2000; Bodelier and Laanbroek, 2004) as well as potassium (Zheng et al. 2008) on growth and activity of MOB have been shown. For nitrogen, this can even by an indirect effect by stimulating N-limited methanogens (Liu et al. 2011). However, the correlations with Na, Mg, Mn, and Cl can only be explained as essential elements for growth, but as yet this has not been demonstrated in soils.

Important for greenhouse gas emissions is the striking difference of the functional and structural response of the MOB community throughout the flooding gradient. Frequent exposure to flooding or permanent flooded conditions in the investigated riparian floodplain leads to a community that immediately responds to methane availability with very high activity levels even higher than in rice field soil (Eller and Frenzel 2001; Kruger and Frenzel 2003; Shrestha et al. 2010) and landfills (Kumaresan et al. 2009) and in the same range as lake sediments (Eller et al. 2005). The oxidation potentials measured in LL and PM exceeded the methane production potentials measured in the same sites by a factor of four to five while also the lag phases in LL for methane production were 10–15 d as compared to 10–20 h for oxidation (Kemnitz et al. 2004). Hence, on the basis of the community potential a history of frequent flooding will lead to a methane oxidation potential large enough to mitigate the methane produced. However, the potentials were measured *in vitro* and in the intact soil physical factors may limit methane oxidation by restricting oxygen entry into the soil. Nevertheless, the biological potential is there to deal with the methane produced but this is largely restricted to a small subgroup of the methanotrophic community and predicting responses to, for example, climate change will necessitate knowledge on the traits of these microbes.

The flooding regime and history in the riparian area studied has led to a very distinct distribution pattern of the MOB community in terms of activity, abundance, and diversity. Although both type I and II MOB were detected by using 16S rRNA PCR-DGGE, the distribution of type specific PLFA clearly demonstrated type I MOB to be generally present and dominant throughout the flooding gradient. This was very different for type II MOB which were only detectable using PLFA in the PM site, where they even outnumbered type I in the deeper layers. However, even in the PM site, the majority of the methane consumed is due to type I species (*Methylobacter/Methylosarcina*) demonstrated by using SIP-PLFA. Type II (*Methylocystis*) MOB are abundant at the PM site but contribute only in a minor way to the activity. Several studies have put forward preferred conditions for growth and activity of type I and II MOB (see Conrad 2007; Semrau et al. 2010) of which high methane concentrations for growth of type II MOB was the most common one (e.g., Henckel et al. 2000). However, using activity proxies (i.e., mRNA; Chen et al. 2007; Abell et al. 2009) or SIP DNA or RNA (Nercessian et al. 2005); (Cebron et al. 2007; Dumont et al. 2011) or SIP–PLFA (Knoblauch et al. 2008) it has been demonstrated that type I MOB are the dominant active MOB in landfill soils, lake sediments, as well as in arctic wetland soils, all being high-methane environments. Hence, this very common statement is generally too crude and the ecology of MOB is a bit more differentiated and diverse, like, for example, the fact that some species can grow facultative on methane or acetate or ethanol (Belova et al. 2011, Im et al. 2011) of which representatives of the genus *Methylocella* cannot be detected by the primers used in this study (Dedysh et al. 2005). What obviously is emerging is the slow reaction of type II MOB to “obvious” good conditions. In a study performed at the LL site, it was demonstrated that when provided with methane, type II MOB had slower growth rates that may have been connected to the lower mRNA transcripts per cell (Steenbergh et al. 2010). Similar results have been observed for lake sediments (Dumont et al. 2011) and rice soils (Krause et al. 2010). Hence, if generalizing at all the r- and k-strategist (Dobzhansky 1950; Golovlev 2001) concept may apply to the ecological strategies of the methanotrophs observed in our soils (Steenbergh et al. 2010). Type I MOB also have been shown to be the active species *in situ* on rice roots and increase their numbers accordingly (Qiu et al. 2008; Shrestha et al. 2010). However, when inoculated into sterile rice soil, type II MOB increased rapidly reaching levels comparable to the total bacterial numbers in nonsterile systems (Ho et al. 2011). Hence, in these microbe free habitats they perform like type I and should be characterized more like “ruderals” occupying open niches while type I's reacts on availability of substrate also in situations of competition with other microbes. Hence, the functional classification framework developed for plants (competitors-stress-tolerators-ruderals, C-S-R) by Grime (1979) may be more applicable to the ecological strategies of MOB than the r–k scheme. The persistence of high numbers of type II MOB in many soils (see Semrau et al. 2010), combined with their presence in acid habitats (Dedysh 2009) as well as substrate versatility (Belova et al. 2011) supports their placement in the ruderal-stress tolerator corner of the Grime triangle.

Remarkably, on the basis of PLFA type II could not be detected in the ML and HL samples and in LL only after incubation. This would be one of the very rare cases that type I outnumber type II in neutral soils. For the samples analyzed in this study, we did not perform QPCR because by the time the method was established in our laboratory the extracted DNA was too old to be processed. However, in a later study performed only in the LL site QPCR was performed using the *pmoA* gene as target (Steenbergh et al. 2010). The *in situ* cell numbers for type II were one order of magnitude higher than type I which is in sharp contrast with the PLFA data. The explanation may be that the cell numbers (see Fig. 5) were calculated based on the lipid C18:1ω8c, which occurs in *Methylocystis* sp. and *Methylosinus trichosporium* (Bodelier et al. 2009) but not in *Methylosinus sporium* as well as *Methylocapsa.* Very recently, a *Methylocystis* species was isolated which also does not possess C18:1ω8c (Im et al. 2011). Hence, there are a quite a few type II candidates that can be detected using *pmoA* gene based methods but not using the specific PLFA. However, assuming that the incorporation of ^13^CH_4_ into other lipids associated with type II MOB (e.g. C18:1ω7c) is caused by incorporation of type I MOB also containing larger amounts of C18:1ω7c (Bodelier et al. 2009) and the failure of obtaining PCR products in ML and HL points into the direction that type II abundance was indeed low, which is remarkable. However, the population starts to develop upon incubation of ML soil as demonstrated by PCR-DGGE that is not reflected in the PLFA. The community profile is highly similar to the one from the LL and the PM site and one additional species is appearing compared to the *in situ* soil profile (Fig. 8). This is in sharp contrast with type I where the profile after incubation shows a much higher diversity in HL and ML as compared to LL and PM (Fig. 7). Apparently more species have the opportunity to proliferate in the soils with low numbers of MOB and with an inactive community. This again demonstrates that type I MOB can react fast in terms of consumption of methane and growth out of a situation of substrate deprivation. However, they do this in soil slurries but when diluted first as is the case in the MPN counting procedure, than only the lowest dilutions display growth in incubations of 4 weeks in the HL and ML soils. Judging from the PLFA cell numbers, type I MOB are there but they do not become active in the higher MPN dilutions, suggesting density dependent activation of activity and growth. However, cultivation biases as the consequence of the media and incubation conditions used cannot be excluded as has been demonstrated (Bussmann et al. 2004).

## Synthesis

It is obvious from the present study that flooding history is reflected in the functional and structural response of the aerobic methanotrophic community in the investigated floodplain soil. More frequent flooding lead to drastic increase in the activity as well as numbers of methanotrophs as well as to an increased response to the availability of methane. Soil-physico chemistry (moisture, organic matter, conductivity) correlated strongly with methane oxidation activity that was carried out by type I MOB. Combining PCR-DGGE and SIP-PLFA profiles demonstrates that the detected MOB are also the active ones. This fact indicates that despite the extensive developments of the molecular methods to assess MOB community composition since the time that this study was carried out, that the approach and methods used do detect the dominant active MOB community members. These MOB seem to display different ecological strategies. To which extend the ecological and physiological characteristics of these species determine the oxidation of methane and hence the flux of methane out of the investigated floodplain remains to be studied. In light of climate change, however, we can conclude that increased flooding of soils will activate methanotrophic communities that have at least the potential to mitigate possible enhanced methane production.

## Appendix

**Table A1:** Physico chemical characteristics of the sampling locations at “Ewijkse Waard” and “Oude Waal” near Nijmegen (The Netherlands) sampled December 13 1999

Location/soil layer	PH H_2_O	Org. matter %	% CaCO_3_	% Clay	% Silt	% sand	Conductivity (S)
HL (0-5cm)	7.58 ± 0.05	9.58 ± 0.75	7.83 ± 0.29	17.51 ± 0.75	36.60 ± 1.70	28.28 ± 2.29	219.75 ± 46.72
(5-10cm)	7.71 ± 0.06	8.14 ± 1.06	8.09 ± 0.51	16.75 ± 1.09	35.45 ± 1.18	30.62 ± 2.16	194.25 ± 20.31
(10-20cm)	7.97 ± 0.02	6.59 ± 0.87	9.32 ± 0.33	17.26 ± 0.32	36.06 ± 0.71	29.92 ± 1.06	175.00 ± 15.08
ML (0-5cm)	7.60 ± 0.03	10.56 ± 0.32	7.75 ± 0.27	28.27 ± 0.53	51.38 ± 0.28	12.11 ± 0.55	219.50 ± 31.46
(5-10cm)	7.74 ± 0.02	9.60 ± 0.34	6.95 ± 0.16	31.54 ± 0.00	57.46 ± 0.00	7.11 ± 0.00	193.25 ± 19.59
(10-20cm)	7.72 ± 0.02	10.50 ± 0.23	7.56 ± 0.14	33.61 ± 0.49	59.71 ± 0.12	6.98 ± 0.16	201.25 ± 23.29
LL (0-5cm)	7.76 ± 0.01	7.41 ± 0.70	7.59 ± 0.46	10.15 ± 1.83	20.78 ± 3.54	56.82 ± 5.45	265.25 ± 24.66
(5-10cm)	7.81 ± 0.02	8.40 ± 0.98	7.51 ± 0.43	16.00 ± 2.80	31.08 ± 5.44	44.14 ± 7.51	288.00 ± 26.73
(10-20cm)	7.86 ± 0.02	12.08 ± 1.42	8.54 ± 0.69	26.56 ± 0.39	51.77 ± 0.57	16.50 ± 0.35	257.75 ± 40.68
PM (0-5cm)	7.28 ± 0.06	20.22 ± 3.52	1.56 ± 1.21	ND	ND	ND	312.00 ± 110.31
(5-10cm)	6.91 ± 0.36	20.18 ± 2.63	0.33 ± 0.53	ND	ND	ND	593.00 ± 131.52
(10-20cm)	7.60 ± 0.17	12.77 ± 4.76	2.04 ± 1.58	26.56 ± 0.39	57.82 ± 8.58	14.32 ± 4.71	523.00 ± 119.30

**Table A2:** Physico chemical characteristics of the sampling locations at “Ewijkse Waard” and “Oude Waal” near Nijmegen (The Netherlands) sampled December 13 1999. HL = high level at the river levee at Ewijk,: ML = Intermediate level and LL = low level. PM = Permanent marshland at Oude Waal

Location/soil layer	Total P mg.kg^−1^	Total N mg.kg^−1^	Na mg.kg^−1^	K mg.kg^−1^	Mg mg.kg^−1^	NH_4_^+^ mg.kg^−1^	Fe mg.kg^−1^	P mg.kg^−1^	SO_4_^2^-mg.kg^−1^	NO_3_-mg.kg^−1^	Cl^−^mg.kg^−1^
HL (0-5cm)	1125 ± 95	3627 ± 302	17.57 ± 3.05	54.17 ± 13.14	117 ± 11	8.08 ± 0.39	0.77 ± 0.05	3.65 ± 0.67	138 ± 30	58.54 ± 11.90	6.41 ± 3.51
(5-10cm)	1050 ± 143	3255 ± 524	19.53 ± 3.25	30.06 ± 10.41	97 ± 11	5.31 ± 0.59	0.75 ± 0.10	1.80 ± 0.56	136 ± 3	45.81 ± 7.16	3.89 ± 0.46
(10-20cm)	845 ± 73	2522 ± 201	20.17 ± 2.18	20.11 ± 4.89	82 ± 10	2.66 ± 0.36	0.67 ± 0.15	0.52 ± 0.22	122 ± 19	31.38 ± 5.21	4.81 ± 0.46
ML (0-5cm)	1894 ± 60	4185 ± 37	33.15 ± 3.16	48.14 ± 4.10	151 ± 8	6.08 ± 0.08	0.57 ± 0.09	3.10 ± 0.25	175 ± 41	52.12 ± 8.47	8.01 ± 4.57
(5-10cm)	2048 ± 40	3427 ± 139	47.05 ± 3.89	23.72 ± 0.66	155 ± 11	2.80 ± 0.41	0.57 ± 0.09	2.87 ± 0.30	178 ± 24	26.33 ± 7.02	5.04 ± 0.53
(10-20cm)	2607 ± 89	3288 ± 74	52.22 ± 4.03	16.22 ± 1.83	170 ± 4	1.75 ± 0.24	0.65 ± 0.10	3.32 ± 0.31	223 ± 39	19.44 ± 4.13	7.55 ± 4.12
LL (0-5cm)	1535 ± 75	1891 ± 180	24.91 ± 4.01	41.34 ± 6.18	81 ± 7	3.22 ± 0.77	0.52 ± 0.05	1.34 ± 0.10	165 ± 43	32.54 ± 13.32	9.62 ± 7.12
(5-10cm)	1942 ± 269	2186 ± 260	42.60 ± 7.70	35.55 ± 5.20	93 ± 7	1.75 ± 0.35	0.50 ± 0.12	1.58 ± 0.07	279 ± 83	26.46 ± 3.50	17.86 ± 10.49
(10-20cm)	2087 ± 85	2683 ± 48	61.68 ± 4.91	52.94 ± 1.87	120 ± 7	2.59 ± 0.52	0.45 ± 0.17	1.85 ± 0.09	352 ± 23	19.83 ± 2.22	13.25 ± 10.01
PM (0-5cm)	1297 ± 210	7834 ± 1534	99.36 ± 24.58	88.32 ± 31.30	220 ± 73	9.22 ± 2.69	0.71 ± 0.23	0.31 ± 0.40	ND	18.43 ± 16.98	187.19 ± 92.08
(5-10cm)	1311 ± 275	7825 ± 978	100.61 ± 4.09	45.44 ± 5.88	241 ± 66	5.63 ± 1.03	0.62 ± 0.15	0.08 ± 0.03	ND	52.64 ± 18.13	112.43 ± 18.72
(10-20cm)	1083 ± 419	4677 ± 1855	64.21 ± 10.94	31.48 ± 8.96	144 ± 30	5.49 ± 1.91	0.62 ± 0.10	0.05 ± 0.02	ND	41.23 ± 17.72	69.84 ± 21.043

**Table A3:** ANOVA analyses of the effects of flooding plain elevation (i.e. site), depth in soil profile and season on initial- and induced methane oxidation activity, most probable numbers of MOB, abundance of MOB-specific PLFA *in situ* and *in vitro*, root biomass and soil moisture content. All variables were LN transformed to meet the assumptions for ANOVA analyses. For all samples n = 4. P values in bold are statistically significant (p < 0.05)

	Df	F	P	Nature of effect
Initial CH_4_ oxidation				
Site (St)	3	11.6803	*0.000000*	+ with flooding
Depth (Dp)	2	4.501	*0.013324*	− in deeper layers
Season (Sn)	2	6.861	*0.001583*	+ in winter
St*Dp	6	3.236	*0.005870*	
St*Sn	6	6.683	*0.000005*	
Dp*Sn	4	0.415	0.797660	
St*Dp*Sn	12	0.591	0.845338	
Induced CH_4_ oxidation				
Site (St)	3	21.15	*0.000000*	+ with flooding
Depth (Dp)	2	3.98	*0.021548*	− in deeper layers
Season (Sn)	2	18.73	*0.000000*	+ in winter
St*Dp	6	6.41	*0.000009*	
St*Sn	6	0.0930	*0.000223*	
Dp*Sn	4	0.76	0.553286	
St*Dp*Sn	12	1.31	0.224434	
Most probable numbers				
Site (St)	3	112.03	*0.000000*	+ with flooding
Depth (Dp)	2	2.27	0.108008	
Season (Sn)	2	17.37	*0.000000*	+ winter
St*Dp	6	1.30	0.264698	
St*Sn	6	11.86	*0.000000*	
Dp*Sn	4	0.34	0.853292	
St*Dp*Sn	12	0.45	0.938636	
Root dry matter				
Site (St)	3	23.8344	*0.000000*	+ with flooding
Depth (Dp)	2	23.8649	*0.000000*	− in deeper layers
Season (Sn)	2	0.3203	0.726677	
St*Dp	6	5.1160	*0.000126*	
St*Sn	6	1.8691	*0.000126*	
Dp*Sn	4	1.7866	0.137343	
St*Dp*Sn	12	0.8761	0.573337	
Moisture%				
Site (St)	3	978.8	*0.000000*	+ with flooding
Depth (Dp)	2	87.4	*0.000000*	− in deeper layers
Season (Sn)	2	36.5	*0.000000*	+ winter
St*Dp	6	15.3	*0.000000*	
St*Sn	6	19.5	*0.000000*	
Dp*Sn	4	0.4	0.793398	
St*Dp*Sn	12	1.8	0.053910	
*C16:1ω5t in situ*				
Site	3	50.287	*0.000000*	+ with flooding
Depth	2	12.123	*0.000100*	− in deeper layers
Site*Depth	6	2.245	0.061620	
*C16:1ω8c in situ*				
Site	3	128.4019	*0.000000*	+ with flooding
Depth	2	20.36	*0.000001*	− in deeper layers
Site*Depth	6	7.3189	*0.000040*	
*C16:1ω5t in vitro*				
Site	3	32.769	*0.000000*	+ with flooding
Depth	2	8.379	*0.001738*	− in deeper layers
Site*Depth	6	5.268	*0.001380*	
*C16:1ω8c in vitro*				
Site	3	22.6195	*0.000000*	+ with flooding
Depth	2	4.6893	*0.019095*	− in deeper layers
Site*Depth	6	4.5852	*0.003104*	

**Table A4:** Spearman Rank order correlation coefficients of measured variables in samples collected in December. Significant correlations are indicated in bold italics. For all values n = 48

Variables	Initial CH_4_ oxidation	Induced CH_4_ oxidation	Most probable numbers
Moisture %	***0.8732***	***0.8549***	***0.8512***
Conductivity	***0.7755***	***0.6285***	***0.7357***
Org. matter %	***0.6057***	***0.6558***	***0.5480***
pH H_2_O	***−0.4103***	***−0.6543***	***−0.3103***
CaCO_3_%	***−0.6556***	***−0.6588***	***−0.5777***
Total P	0.1237	−0.0818	0.1172
Total N	***0.3224***	***0.5895***	0.2513
SO_4_	***0.6870***	0.2341	***0.6118***
P	***−0.5798***	***−0.4043***	***−0.5678***
Na	***0.7931***	***0.6107***	***0.7613***
K	***0.4566***	***0.6115***	***0.4595***
Mg	***0.4620***	***0.5190***	***0.4293***
NO_3_	−0.1646	0.1601	−0.2424
NH_4_^+^	0.1939	***0.5560***	0.1200
Fe	−0.2339	0.0232	−0.2736
Mn	***0.5587***	***0.5761***	***0.4450***
Zn	0.1740	0.1955	0.046
Cu	***−0.5703***	***−0.6363***	***−0.5454***
Cl	***0.8033***	***0.5756***	***0.7081***

**Table A5:** Most similar sequences according to BLAST comparison of sequenced DGGE bands. Nearest cultured representative is also given

Band (bp)	Closest relative (accession number)	Coverage	Max identity	Taxonomy	Habitat
BMHL3 (448) DGGE band 15 (JN254762)	Uncultured gamma proteobacterium partial 16S rRNA gene, DGGE band BDPM9. (AJ831503)	98%	98%	Type Ia, γ	Floodplain soil
	Methylobacter sp. T20 (AF131868)	98%	98%	Type Ia, γ	Swamp soil
BMHL22 (447) DGGE band 35 (JN254763)	Methylobacter sp. LW12 16S ribosomal RNA gene (AY007295)	99%	97%	Type Ia, γ	Lake sediment
BMHL23 (448) DGGE band 36 (JN254764)	Uncultured type I methanotroph clone site1–39 16S ribosomal RNA gene, partial sequence (EF101324)	99%	97%	Type Ia, γ	Lake sediment l
	Methylobacter sp. T20 (AF131868)	99%	96%	Type Ia, γ	Swamp soil
BMHL24 (453) DGGE band 30 (JN254765)	Methylobacter luteus NCIMB 11914 16S ribosomal RNA gene (AF304195)	99%	98%	Type Ia, γ	Landfill soil
BMHL25 (454) DGGE band 31 (JN254766)	Uncultured bacterium clone SIP CM44 16S ribosomal RNA gene, partial sequence (EU131042).	99%	99%	Type Ia, γ	Coal mine soil
	Methylomicrobium agile strain ATCC 35068 16S ribosomal RNA gene (EU144026)	99%	97%	Type Ia, γ	Mineral soil
BMLL11(449) DGGE band 5 (JN254767)	Uncultured bacterium clone 1H_73 16S ribosomal RNA gene, partial sequence (AY546504)	99%	99%	Type Ia, γ	Soda Lake sediment
	Methylobacter tundripaludum 16S ribosomal RNA (AJ414655)	99%	98%	Type Ia, γ	Arctic soil
BMLL12 (451) DGGE band 4 (JN254768)	Uncultured gamma proteobacterium partial 16S rRNA gene, DGGE band BDPM9 (AJ831503).	98%	99%	Type Ia, γ	Floodplain soil
	Methylobacter tundripaludum 16S ribosomal RNA (AJ414655)	99%	98%	Type Ia, γ	Arctic soil
BMLL34 (453) DGGE band 21 (JN254769)	Methylobacter sp. LW12 16S ribosomal RNA gene (AY007295)	99%	98%	Type Ia, γ	Lake sediment
BMLL49.1 (438) DGGE band 49 (JN254770)	Uncultured type II methanotroph clone CHOII-7 16S ribosomal RNA gene, partial sequence (HM209451).	99%	99%	Type II, α	Landfill cover soil
	Methylosinus trichosporium partial 16S rRNA gene, strain KS24b(AJ458496)	99%	99%	Type II, α	Lake sediment
BMLL4 (454) DGGE band 9 (JN254771)	Methylobacter luteus NCIMB 11914 16S ribosomal RNA gene (AF304195)	99%	98%	Type Ia, γ	Landfill soil
BMLL6 (453) DGGE band 11 (JN254772	Uncultured gamma proteobacterium isolate DGGE gel band TypeI-06 16S ribosomal RNA gene, partial sequence (EU170106)	99%	96%	Type I, γ	Landfill soil
BMLL32 (454) DGGE band 24 (JN254773)	Uncultured bacterium clone 1H_73 16S ribosomal RNA gene, partial sequence (AY546504)	98%	98%	Type Ia, γ	Soda lake sediment
	Methylobacter tundripaludum 16S ribosomal RNA (AJ414655)	99%	98%	Type Ia, γ	Arctic soil
BMPM17(454) DGGE band 1 (JN254774)	Uncultured gamma proteobacterium partial 16S rRNA gene, DGGE band BDPM4 (AJ831498)	99%	98%	Type Ia, γ	Floodplain soil
	Methylobacter sp. LW1 16S ribosomal RNA gene (AF150784)	99%	96%	Type Ia, γ	Lake sediment
BMPM18(448) DGGE band 2 (JN254775)	Uncultured gamma proteobacterium partial 16S rRNA gene, DGGE band BDPM4 (AJ831498)	99%	99%	Type Ia, γ	Floodplain soil
	Methylobacter sp. LW1 16S ribosomal RNA gene (AF150784)	99%	96%	Type Ia, γ	Lake sediment
BMPM19(448) DGGE band 3 (JN254776)	Uncultured gamma proteobacterium partial 16S rRNA gene, DGGE band BDPM4 (AJ831498)	99%	99%	Type Ia, γ	Floodplain soil
	Methylobacter sp. T20 (AF131868)	99%	96%	Type Ia, γ	Swamp soil
BMPM35(447) DGGE band 20 (JN254777)	Uncultured type I methanotroph clone site1–39 16S ribosomal RNA gene, partial sequence (EF101324).	99%	99%	Type Ia, γ	Lake sediment
	Methylobacter tundripaludum 16S ribosomal RNA (AJ414655)	99%	98%	Type Ia, γ	Arctic soil
BMPM46(440) DGGE band 46 (JN254778)	Methylocystis sp. L32 partial 16S rRNA gene, clone L32 (AJ831522)	100%	99%	Type II, α	Floodplain soil
BMPM55–1 (436) DGGE band 55 (JN254779)	Methylocystis sp. L32 partial 16S rRNA gene, clone L32 (AJ831522)	99%	99%	Type II, α	Floodplain soil
BMPM60–2 (436) DGGE band 60 (JN254780)	Methylocystis sp. H4 partial 16S rRNA gene, clone H4. (AJ831519)	99%	98%	Type II, α	Floodplain soil
BMPM63–2 (435) DGGE band 63 (JN254781)	Methylocystis sp. L32 partial 16S rRNA gene, clone L32 (AJ831522)	99%	99%	Type II, α	Floodplain soil

**Table A6:** Spearman Rank order correlation coefficients of methane oxidation activity and numbers with MOB specific PLFA and root biomass in soil samples collected in May. Significant correlations are indicated in bold italics. For all values n = 48

Variables	Initial Oxidation	Induced oxidation	MPN	Roots biomass	C16:1ω5t *in situ*	C16:1ω8c *in situ*	C16:1ω5t *in vitro*
Initial Oxidation							
Induced oxidation	***0.4876***						
MPN	***0.8008***	***0.3556***					
Roots biomass	***0.5513***	***0.4169***	***0.3705***				
C16:1ω5t *in situ*	***0.3281***	***0.3469***	0.0933	***0.4687***			
C16:1ω8c *in situ*	***0.6853***	***0.3434***	***0.6681***	***0.5381***	***0.6533***		
C16:1ω5t *in vitro*	***0.4661***	***0.3376***	0.2246	***0.4327***	***0.5458***	***0.4948***	
C16:1ω8c *in vitro*	***0.6547***	***0.4457***	***0.6017***	***0.2348***	0.2307	***0.5474***	***0.7638***

**Table A7:** Spearman Rank order correlation coefficients of variables measured on samples collected in December, May and September. Significant correlations are indicated in bold italics. For all values n = 144

Variables	Initial Oxidation	Induced oxidation	MPN	Roots biomass
Initial Oxidation				
Induced oxidation	***0.5791***			
MPN	***0.6915***	***0.3677***		
Roots biomass	***0.3433***	***0.4188***	***0.1789***	
Moisture	***0.7887***	***0.5346***	0.0933	***0.3431***
